# Diving into commercial cellulase formulations for circular polyester/cotton separation through targeted depolymerization of cotton

**DOI:** 10.3389/fbioe.2025.1632772

**Published:** 2025-09-19

**Authors:** Jeannie Egan, Michael Barta, Patrick Pointner, Birgit Herbinger, Judith Rudolf-Scholik, Agnes Gruenfelder, David Lilek, Thomas Rosenau, Georg M. Guebitz, Christian B. Schimper

**Affiliations:** ^1^ Josef Ressel Centre “Recovery Strategies for Textiles”, University of Applied Sciences Wiener Neustadt, Biotech Campus Tulln, Tulln an der Donau, Austria; ^2^ Department of Natural Sciences and Sustainable Resources, Institute of Chemistry of Renewable Resources, BOKU University, Tulln an der Donau, Austria; ^3^ Department of Agricultural Sciences, Institute of Environmental Biotechnology, BOKU University, Tulln an der Donau, Austria

**Keywords:** cellulase, polyester/cotton separation, activity assays, bulk hydrolysis, enzyme technology

## Abstract

The rapid growth of the textile industry, driven by fast fashion trends, has significantly increased textile waste, particularly blends of polyester (PET) and cotton fibers, and efficient recycling of blended textiles requires effective fiber separation methods, yet current strategies face substantial limitations. This study investigates the performance of commercially available cellulase formulations for enzymatic depolymerization of cotton and suggests an optimized activity pattern for novel reactor systems for recycling of PET/cotton blends. Thirty-five cellulase formulations intended for biopolishing, stonewashing, or biomass degradation were extensively characterized through biochemical assays (protein quantification, reducing sugar content, SDS-PAGE, and enzyme-specific activity assays). The formulations were further categorized according to their physical state, optimal pH and temperature class, and use case (biopolishing, stonewashing, and degradation), and trends were observed between the categories. Ten formulations were selected for further practical evaluation in a controlled reactor system to determine cotton removal efficacy from a blended PET/cotton fabric based on cotton weight loss. Enzyme activity assays did not correlate directly with reactor performance, suggesting that conventional cellulase assays may inadequately predict real-world separation efficiency. Protein efficiency was introduced as a critical metric for evaluating enzyme efficacy and economic viability. Here this variable was defined as a specific activity, relating the fiber separation efficiency in the reactor to the protein content of the formulation to indicate how effective the proteins were at separation. Formulations exhibiting balanced high performance in both weight loss and protein efficiency predominantly contained high proportions of endoglucanase activity, supported by moderate cellobiohydrolase and minor beta-glucosidase activities.

## Graphical Abstract

**Figure:**
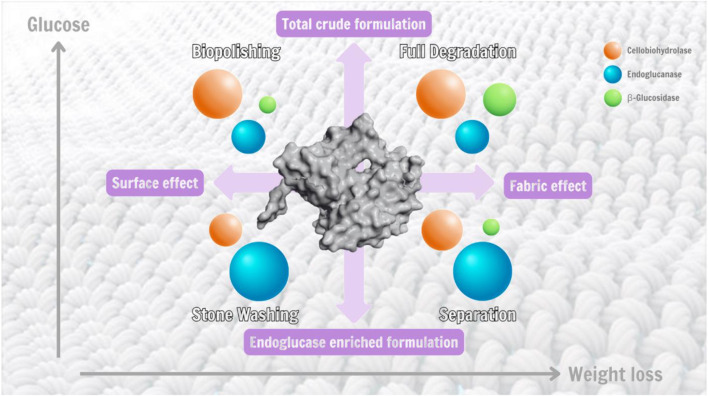
Shows illustration of the endoglucanase (blue), cellobiohydrolase (orange), and beta-glucosidase (green) components in each cellulase formulation for different use cases and the relative characteristics of each.

## 1 Introduction

Fast fashion and excessive consumption have made textile waste accumulation a prevalent and time-sensitive issue. The textile industry follows a microtrend model that requires expedited linear production pathways to meet increased demand (3.7 kg/person consumed in 1950 versus 11.1 kg/person in 2007 and 16 kg/person in the EU in 2020 (Pensupa et al., 2017; European Environment Agency, 2024). Textile production has doubled in the last 13 years, whereas the amount of time that clothing is worn before disposal has decreased by 40% (Piribauer et al., 2021; Wojnowska-Baryła et al., 2022). These trends have led to a large amount of textile waste, which is mostly landfilled or incinerated (Wojnowska-Baryła et al., 2022). The European Green Deal (European Commission, 2022) has established objectives with a set endpoint of 2030 that includes the sustainable management of textiles in response to the fast fashion era.

Cotton and polyester hold the largest market share of textile fibers where—of the 124 million tons of fiber production globally—PET represents the largest volume at 57%, while cotton and other cellulosic fiber types (viscose, lyocell, *etc.*) represent around 31% based on 2023 estimates (Textile Exchange, 2024). Though textile recycling strategies for both isolated cotton and polyester textile waste are known, only 12% of polyester (PET) fibers are made from recycled PET (predominantly starting as plastic bottles) (Textile Exchange, 2024), and less than 1% of all fibers find their way into new garments again (Ellen MacArthur Foundation, 2017; McKinsey and Company, 2022). An additional challenge to recycling is introduced when fiber types are blended, requiring mostly chemical or biochemical separation technologies to generate clean waste streams of each fiber as a precursor to the actual recycling. Separation methodologies like acid hydrolysis (Bragd et al., 2024; Binczarski et al., 2024), ionic liquid dissolution (Jeihanipour et al., 2010), and hydrothermal processing (Speight et al., 2020; Yee et al., 2020; Barla et al., 2022) have been investigated and are currently being upscaled. The present research focuses on the separation of PET/cotton fabrics using enzymatic hydrolysis of cotton by cellulases, which is generally known to be a possible route that has not been explored on an industrial scale yet (Gritsch et al., 2023).

Cellulases are a class of hydrolytic enzymes that naturally work on cellulose, breaking it down from its polymeric structure into monosaccharides. This enzyme category generally has both oxidizing enzymes—like polysaccharide monooxygenases (PMOs, (Beeson et al., 2015; Bissaro et al., 2018)) or lytic PMOs (LPMOs, (Frommhagen et al., 2015; Sulaeva et al., 2024)) and hydrolytic enzymes. To completely degrade cellulose, a combination of three main types of hydrolytic enzymes is required: endoglucanases, cellobiohydrolases (CBH), and beta-glucosidases (BG). Endoglucanases are bulk cellulose hydrolysis enzymes, which cleave cellulose polymer chains at beta-glycosidic linkages randomly along the backbone (in amorphous regions) to create two separate polymer chains of varying lengths (Srivastava et al., 2018). CBHs degrade the cellulose from the ends, known to work in highly ordered crystalline areas of cellulose, to release a disaccharide cellobiose unit (Sajith et al., 2016; Singh et al., 2016). The released cellobiose is broken down into two separate glucose units by BGs (Singharia et al., 2017). Due to the high selectivity of cellulase enzymes, this process can be applied to PET/cotton blended textile substrates, where cotton is enzymatically depolymerized while the polyester remains unaffected and suitable for processing into high-quality granulate using conventional PET recycling machines (Gritsch et al., 2023).

In the textile industry, cellulases are already used as a sustainable alternative to conventional environmentally harmful chemicals in fabric preparation to impart unique effects for aesthetic purposes. For example, stonewashing of denim can be carried out with cellulases, which are known to peel back layers of the fiber surface to release indigo dye molecules without excessive damage to the fabric when the process conditions are set correctly. Additionally, biopolishing is another process that exploits cellulases to enhance fabric quality by smoothing the textile surface to reduce pilling and improve color brightness through fiber cleavage (Kuhad et al., 2011; Sharma et al., 2016). Further, for utilization of cellulose-containing organic materials in biorefining processes, formulations have emerged that are used for efficient bioconversion of various cellulosic biomass sources into sugar that is used as a platform chemical. These formulations sometimes include oxidizing cellulases for synergistic degradation effects (Bissaro et al., 2018), but these components will not be a focus of the present work.

Since the three main classes of hydrolytic cellulase enzymes are acting synergistically, commercial enzyme formulations can be optimized for different purposes. There are variations in the expression and purification systems between different manufacturers or products, leading to diverse compositions of cellulase enzymes between commercial formulations. Further, additional components of these formulations vary with use case and shelf stability requirements where formulations may contain added stabilizers, sugars, or buffers that allow for different beneficial properties.

In this research, commercial cellulase formulations curated for the purposes of either biopolishing (BP), stonewashing (SW), or degradation (Deg) were screened using various cellulase-specific activity assays as well as other general analytical techniques to demonstrate and draw conclusions on their use in a new application: removal of cellulose fibers from blended fabrics. To achieve this, relatively simple test procedures that could potentially be carried out prior to application in industry were primarily considered. Data collected from the filter paper, CellG5, Avicel, and beta-glucosidase assays offered both holistic formulation and enzyme-specific information, which was compared across categories to observe trends within and between assays. Some of the formulations were selected for further testing in an internally developed separation methodology to investigate the relationship between assay data and fiber separation efficiency. The observed trends were used to inform conclusions about the important factors that could determine what a theoretical formulation optimized for separation of mixed textile blends would require while internally identifying which commercially available formulations would be useful for future study in the area. Likewise, this knowledge would help industry to select and rapidly characterize commercial formulations for this novel application.

## 2 Materials and methods

### 2.1 Materials

Enzyme formulations were collected from several different global chemical suppliers that promote their formulations for different industrial use cases. Formulations for use in biopolishing and stonewashing and for total degradation in biorefinery systems were selected. To honor their intellectual property, the names of the suppliers and their formulations are not disclosed here. Instead, the focus lays on the screening of a large amount of formulations from different classes and use cases to draw conclusions on general trends in behavior of these formulations.

Therefore, prior to assay screening, formulations were categorized according to their physical state (liquid and solid), optimal pH and temperature conditions, and intended commercial application according to the supplier’s technical data sheet (Table 1). Optimal pH was split into two general subcategories of acidic and neutral, with a pH of 6 being the threshold between the two distinctions. Temperature was also divided into two subcategories with optimal temperature below 45 °C being classified as “cold” and above 45 °C as “warm”. A total of 35 formulations were tested. 17 of these were classified as active formulations and were used for the bulk of this analysis, while 18 were beyond their shelf life at recommended conditions (e.g., 4 °C for liquid formulations, room temperature for solid formulations), so likely showed reduced activity from their most active state (5 showed no activity at all). Long term storage can negatively impact the activity of cellulase formulations (Himmel et al., 2017), but these 18 enzymes were still screened because some activity was identified for most of them and were only analyzed in comparison to the active formulations and were not used in the main analysis presented. Notably, the formulations compiled for this study were randomly labeled as “Cel” and numbered from 1 to 35 (later, 5 were excluded since they did not show any activity). As mentioned earlier, these formulations were taken from different use cases, which were also used as a means of categorization: biopolishing, stonewashing and degradation (for use in biorefineries). After data evaluation from the activity assays, enzymes were further categorized and compared according to their enzyme proportion, or the ratio of enzymes present in the formulation, being called either total crude (TC) or endo-enriched (EG).

**TABLE 1:** Categorization of commercial enzyme formulations by physical state, optimal pH conditions (acid = 4-6, neutral = 6–7), optimal temperature conditions (cold = <45 °C, warm = >45 °C), enzyme proportion, and intended commercial purpose. The rightmost column shows the distribution of 10 formulations selected for reactor testing (foundational formulations were not considered because a foundational study already included information about some of these formulations (Struszczyk et al., 1997)).

Category	Distinction	Beyond shelf life (13 formulations)	Active (17 formulations)	Reactor testing (all Active)
Physical State	Liquid	10	11	6
	Solid	3	6	4
Optimum pH	Acid	7	5	5
	Neutral	6	12	5
Temperature	Cold	5	9	5
	Warm	8	8	5
Use Case	Biopolishing (BP)	6	8	4
	Degradation (Deg)	0	7	4
	Stonewashing (SW)	7	2	2
Enzyme Proportion	Total Crude (TC)	4	6	4
	Endo-Enriched (EG)	9	11	6

The textile fabric used was a white linen woven bedsheet (140 g/m^2^) with a yarn composition of 50% cotton and 50% polyester, supplied by Salesianer Miettex GmbH, Austria. Prior to hydrolysis experiments, all fabrics were washed with 2% on weight of fabric sodium carbonate (technical quality) at 60 °C using a liquor ratio of 1:10, subsequently rinsed 5 times with deionized water, and dried. The cellulose content was confirmed according to ISO 1833–11:2017 using sulfuric acid (ISO 1833-11:2017, 2017).

All other chemicals used in these experiments were of analytical grade and purchased from Sigma-Aldrich, if not mentioned otherwise in the description.

### 2.2 Protein quantification and biochemical assays

#### 2.2.1 Bradford method for protein content analysis

An adapted method of the Bradford assay (Bradford, 1976) was used to quantify protein content in triplicates in each formulation, according to the Olson and Markwell Micro-Bradford method (Olson and Markwell, 2007). Briefly, 100 µL of an enzyme formulation was combined with 150 µL of the Bradford dye reagent and allowed to react for 5 min before spectrophotometric measurement at 595 nm. Enzyme formulations were diluted to different levels (variation between formulations from 10x-2000x dilution) to fit within the range of the prepared calibration curve. For solid formulations, 1 g was dissolved in 10 mL of citric acid buffer (20 mM) then further diluted to fit within the calibration range if necessary. From this stock solution, protein content was measured as described above.

#### 2.2.2 Neocuproine method for reducing sugar analysis

Reducing sugars in each formulation were quantified in triplicate using the neocuproine method (Cavaco-Paulo et al., 1996; Schimper et al., 2006), with adaptations that corresponded with the instruments available. The reaction was carried out using 50 µL of sample, 2 mL of sodium carbonate solution (2% w/v), 5 mL neocuproine reagent, and 10 mL of deionized water in a test tube at 100 °C for 5 min. The samples were diluted before spectrophotometric measurement at 475 nm to match the range of the prepared calibration curve. This method was also used to quantify results during assay screening for the Avicel and filter paper assays.

#### 2.2.3 Filter paper — total cellulase activity

A modified filter paper assay using Whatman No. 1 filter paper (Ghose, 1987) was applied in this work to determine overall formulation activity, quantifying the synergistic action of all three cellulase activities. Filter paper was manually cut to create 10 weight fractions between 0-50 mg substrate which were each mixed with 1 mL citric acid buffer (20 mM) and 100 µL of the enzyme stock solution (0.0327 mg protein/mL) in a 2 mL microcentrifuge tube. The samples were allowed to react in a shaking thermomixer at 50 °C for 60 min (750 rpm), after which 100 µL of sodium carbonate solution (15% w/w) was added to stop the reaction. After centrifugation at 10,000 rpm for 5 min, the supernatant of the resultant solutions was used according to the neocuproine method for reducing sugar quantification. The reactions were measured in triplicate.

#### 2.2.4 KCellG5 — endoglucanase assay

Endoglucanase activity (random chain scission) was quantified in duplicate using the Megazyme KCellG5 assay kit according to the method provided in the kit (Megazyme, 2019), using CellG5 as the substrate. The protocol calls for 0.1 mL of CellG5 to be added to a glass tube and pre-incubated to 50 °C in a water bath before adding 0.1 mL of the appropriately diluted enzyme solution (ranged from 2500x-50,000x) and incubating for 10 min, after which 3 mL of stopping reagent (2% w/v Tris buffer) was added. Then, the samples were measured at 400 nm against a reagent blank and distilled water.

#### 2.2.5 Avicel — cellobiohydrolase assay

Cellobiohydrolase activity—which represents the action of releasing cellobiose from both the reducing and non-reducing ends of the cellulose chain—was measured using Avicel PH-101 as a model crystalline substrate (Wood and Bhat, 1988). 10 different substrate masses in a range between 0-15 g were mixed in 100 mL Schott flasks with 49 mL of buffer solution (20 mM citrate buffer) and 1 mL of enzyme stock solution (2 mg/mL). This mixture was allowed to react in a shaking water bath (200 rpm, with five 5 mm steel balls) at 50 °C for 60 min, when sodium carbonate solution was added to stop the reaction. Each reaction was measured in triplicate using the neocuproine method for reducing sugar quantification on the supernatant.

#### 2.2.6 p-nitrophenyl-beta-D-glucopyranoside — beta-glucosidase assay

Beta-glucosidase activity—the ability to cleave the disaccharide cellobiose to two glucose units—was measured according to an internally adapted method using p-nitrophenyl-beta-D-glucopyranoside (PNP) as a dimeric substrate (Wood and Bhat, 1988). Ten concentrations (0–8 mmol/L) of the PNP substrate were added directly into the wells of a microtiter plate with 10 µL of the enzyme solution (at the predetermined optimal dilution level) and filled up to 50 µL with deionized water and allowed to react in an incubator at 37 °C for 60 min, at which point 200 µL of stop solution (0.4 M Glycine/NaOH, pH 10.4) was added. Each test was measured in triplicate. Samples were photometrically measured at 405 nm, and using a 4-nitrohpenol calibration, the cleaved monosaccharide amount was determined.

#### 2.2.7 Assay analysis

It is understood that the Michaelis-Menten model is not representative of the kinetics in most cellulase systems, namely, those involving a solid substrate and an undefined enzyme cocktail like the filter paper and Avicel assays (Nagl et al., 2022; Olkiewicz et al., 2022), and that other models have been developed that are more illustrative (Levine et al., 2010; Nagl et al., 2021). Despite this, the data was interpreted according to the Michaelis-Menten equation, wherein V_max_ is the limiting rate approached by the system at saturated substrate concentration for a given enzyme concentration, and the Michaelis constant K_m_ is defined as the concentration of substrate at which the reaction rate is half of V_max_. In general, high V_max_ values and low K_m_ values were understood to indicate high activity and high substantivity, respectively (Berg et al., 2006). In the case of the beta-glucosidase assay which uses a liquid substrate and specifically targets the final enzyme in the cellulase hydrolytic cascade, the Michaelis-Menten kinetic data is expected to be representative. Throughout this work, all kinetic constants and discussions of them should therefore be understood as apparent kinetic constants (V_max_^*app*^ and K_m_^*app*^).

### 2.3 Sodium dodecyl sulfate–polyacrylamide gel electrophoresis (SDS-PAGE)

SDS-PAGE was carried out to determine the molecular weight of the protein fractions in the sample (Tschesche, 2011). Samples were prepared using the volume needed for 5 μg of protein, based on the Bradford assay quantification. Denatured protein samples were loaded into the gel, along with 3 µL of marker solution, which served as a reference for the subsequent analysis. Electrophoresis was performed at 35 mA for ∼40 min. Afterwards, the gels were stained with Coomassie Blue solution before scanning using a GS-800 Calibrated Densitometer.

### 2.4 Hydrolysis performance in reactor

Selected enzyme formulations were tested for their potential to remove cotton from a 50/50 PET/cotton blended fabric in an enclosed reactor system with controlled heating and agitation. Temperature and pH were set as a standard pH 6 °C and 60 °C for all tested formulations. The enzyme concentration was set to 2% on weight of fabric (owf), independent of the protein concentration, and the reaction time was set as 4 h. While the dosage recommendations of the suppliers are meant to avoid fabric damage during textile processing (below 1% owf in most cases), the dosage in these experiments was intentionally fixed and set high since the goal is total degradation of the cellulosic substrate. Some samples were further tested at other pH or temperature conditions, discussed later. Cotton hydrolysis was quantified gravimetrically and reported as cotton weight loss. This experiment was carried out once for every hydrolysis experiment described in the work except for Cel 28, which had 10 repetitions.

Selection of enzymes to test in the reactor was based on several characterization factors including intended use case, physical classification (temperature, pH, and solid state), and identification as either total crude (TC) or endo-enriched (EG) based on assay data. Selecting a variety of classifications was important to allow conclusions to be drawn about different categories and their suitability for the rapid removal of cotton from the textile substrate. Enzymes used for reactor testing were selected based on their assay data and to intentionally cover a variety of these classifications.

## 3 Results

### 3.1 Protein and reducing sugar content

**FIGURE 1:**
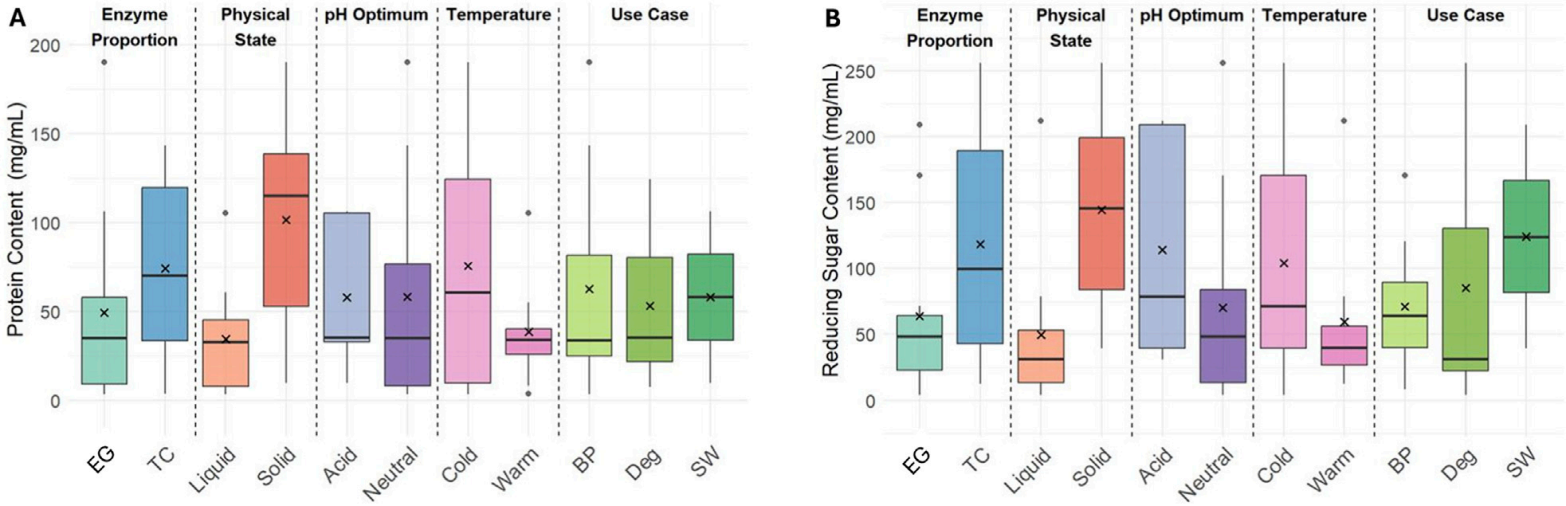
Box plots showing distributions of protein content **(A)** and reducing sugar **(B)** measured by Bradford and neocuproine assays across different enzyme categories (in this plot, the average is marked by an X, the median is the horizontal line in the box, the box corresponds to the interquartile range with 25% above and 25% below the median, the antennas are maximum 1.5 fold of the interquartile distance, and the individual points represent possible outliers).

The Bradford assay was performed on all enzyme formulations to quantify the protein content in the formulations (Figure 1A). A large variety in protein concentration was observed with a range of 3.47–190 mg/mL with an average of 58.1 mg/mL for the active formulations. In general, the enzyme formulations beyond their shelf life had the smallest amount of protein with an average of 7.03 mg/mL (data not shown). The average protein concentration is notably higher in the six solid formulations (101.4 mg/g) compared to liquid ones (34.5 mg/mL), which makes sense because the content of protein in a liquid formulation is, in part, controlled and limited by the relative solubility in the solvent. The average protein content for formulations in both optimal pH categories (neutral and acid) were similar, with neutral having a slightly greater variation. The different temperature class distinctions showed lower protein concentrations in warm formulations and a larger variation in cold formulations. Interestingly, the formulations used for stonewashing had a higher average protein content than the other two use cases. However, the current dataset included only two SW formulations—one with very low protein content and one with very high—limiting the ability to draw a broader conclusion based on this knowledge.

The higher protein content for TC and solid formulations compared to their EG and liquid respective counterparts is to be expected. Continuous fabric processing used during biopolishing requires liquid formulations for automatic dosing of the surface and typically requires acidic pH and elevated temperature for maximum performance. In contrast, enzyme formulations that are effective at low temperatures are preferred in laundry processes, like stonewashing, which aim for low energy consumption. The intended aesthetic effect in stonewashing is controlled by localized abrasion of the fiber surface to release indigo particles from the garment; therefore, EG formulations are often used. In this dataset, there were only two SW formulations: one with very low protein content and one with very high. SW formulations are optimized for abrasion and are typically formulated with low activity to avoid damaged garments through overdosing, but this is not evident for the measured protein content from this data set.

The neocuproine assay was carried out on all formulations to quantify the amount of reducing sugars present as additives in each formulation. Since the filter paper and Avicel assays were quantified using this method, understanding the baseline of reducing sugar content of each individual formulation was necessary. The reducing sugar content in the formulations was found to be in a wide range between 4.23-256 mg/mL, and the data displayed more distinguishing features between the categories than protein content (Figure 1B). TC and solid formulations exhibited significantly higher reducing sugar content than EG and liquid, respectively. The relationship between physical state could indicate that the granulation and stabilization of the enzymes requires agents with reducing sugar additives. Cold and acid formulations showed a higher content and a greater variability than their warm and neutral counterparts. The SW formulations in this dataset also had a higher reducing sugar content, which may have been an artifact of both being solid formulations.

### 3.2 Protein profile via SDS-PAGE

**FIGURE 2:**
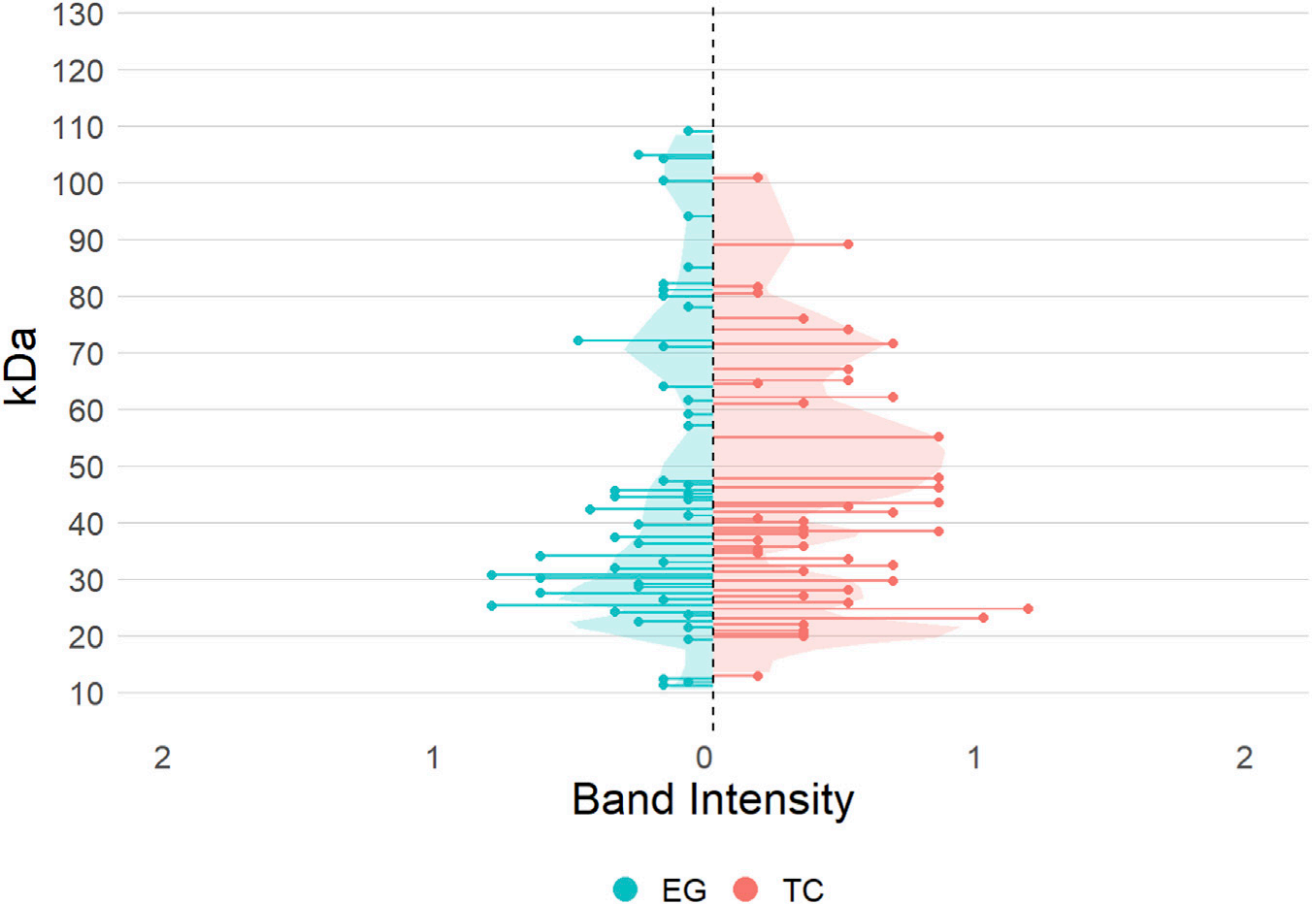
Band intensity averages evaluated from SDS-PAGE data for TC (orange) and EG (blue) categories from all current formulations. Shaded areas under LOESS-smoothed curves (span = 0.2) highlight the overall signal distribution per group across the molecular weight range.

Internally, the bands at different molecular weights (in kDa) were evaluated according to their intensity and categorized into four levels: low, medium, high, and very high (Supplementary Figure S1 in Supplementary). A representation of the bands for each formulation based on this distinction is displayed in Supplementary Figure S2 (Supplementary). Furthermore, the mean intensity of all bands of formulations (grouped by TC and EG distinctions) at the same molecular weight was calculated and plotted for a better understanding (Figure 2). Both formulation types showed only a restricted number of peaks in the range of 75 kDa and above.

TC formulations exhibited two areas with high average intensities: between ∼19-45 kDa and between 58-75 kDa. The band at 37 kDa may be associated with an endoglucanase, as detected by Nisar et al. (2022). As noted by Foody and Tolan (Tolan and Foody, 1999), the high intensities at 57 kDa and 66 kDa could possibly be CBHI and CBHII, respectively (Imangholiloo et al., 2022). However, beta-glucosidases can also exhibit a molecular weight of 57 kDa in its monomeric structure (Mateos et al., 2015). The highest peak was detected at 21 kDa with an average intensity of approximately 1.2 using the perceived band intensity method visualized in Supplementary Figure S2 (Supplementary).

In contrast, EG formulations reached maximum band intensities between 22-31 kDa, so it may be assumed that the bands with very high intensity between 22-31 kDa are specific to endo-enriched formulations. Bands at approximately 21 kDa could possibly be explained by a β-1,4-endoglucanase (Rahman et al., 2014) and bands at 27 kDa by 1,3–1,4-β-D-glucanase (Chen et al., 1997), which have been observed by other groups. Moreover, bands in the range from 19 to 31 kDa may be caused by EGIII and EGV in general (Tolan and Foody, 1999).

Apart from the three classical cellulase components, small molecular weights may include other proteins like carbohydrate binding modules (up to 20 kDa (Shoseyov et al., 2006)) or expansins between 10-30 kDa (Artzi et al., 2016). Higher molecular weights are reported for cellulose-induced proteins (∼30–50 kDa (Jia et al., 2021)) and swollenins with about 50–70 kDa (Wang et al., 2010). Oxidative enzymes have also been reported for cellulase formulations including LPMOs between 15-30 kDa (Srivastava et al., 2022), PMOs between 25-35 kDa (Bulakhov et al., 2018), and cellobiose dehydrogenases (80–100 kDa (Schou et al., 1998)) that support LPMOs. Most of the bands seen in the current SDS-PAGE data could be associated with more than one possible contributing protein deriving from several different origins. Further exploration was determined to be beyond the scope of this work.

#### 3.3 Cellulase Assays

Within this section, all assay results will be discussed in terms of apparent kinetic values based on the Michaelis-Menten model for enzymes [50]. It is well understood that this model is not adequate to fully represent the activity of multicomponent cellulase formulations on a solid substrate (Olkiewicz et al., 2022), like the filter paper or Avicel substrates. There have been other groups that have developed more representative models for cellulase-based activity assessments (Levine et al., 2010; Nagl et al., 2021). Despite this knowledge, one of the objectives of the current work was to generate data that could be relevant in industrial practice, which meant conducting evaluations using simplified and straightforward methods for characterization of cellulase formulations. Though the kinetic values presented for the filter paper and Avicel assays should only be interpreted as apparent values, they can act as a baseline for formulation characterization and comparison to the separation use case investigated here.

**FIGURE 3:**
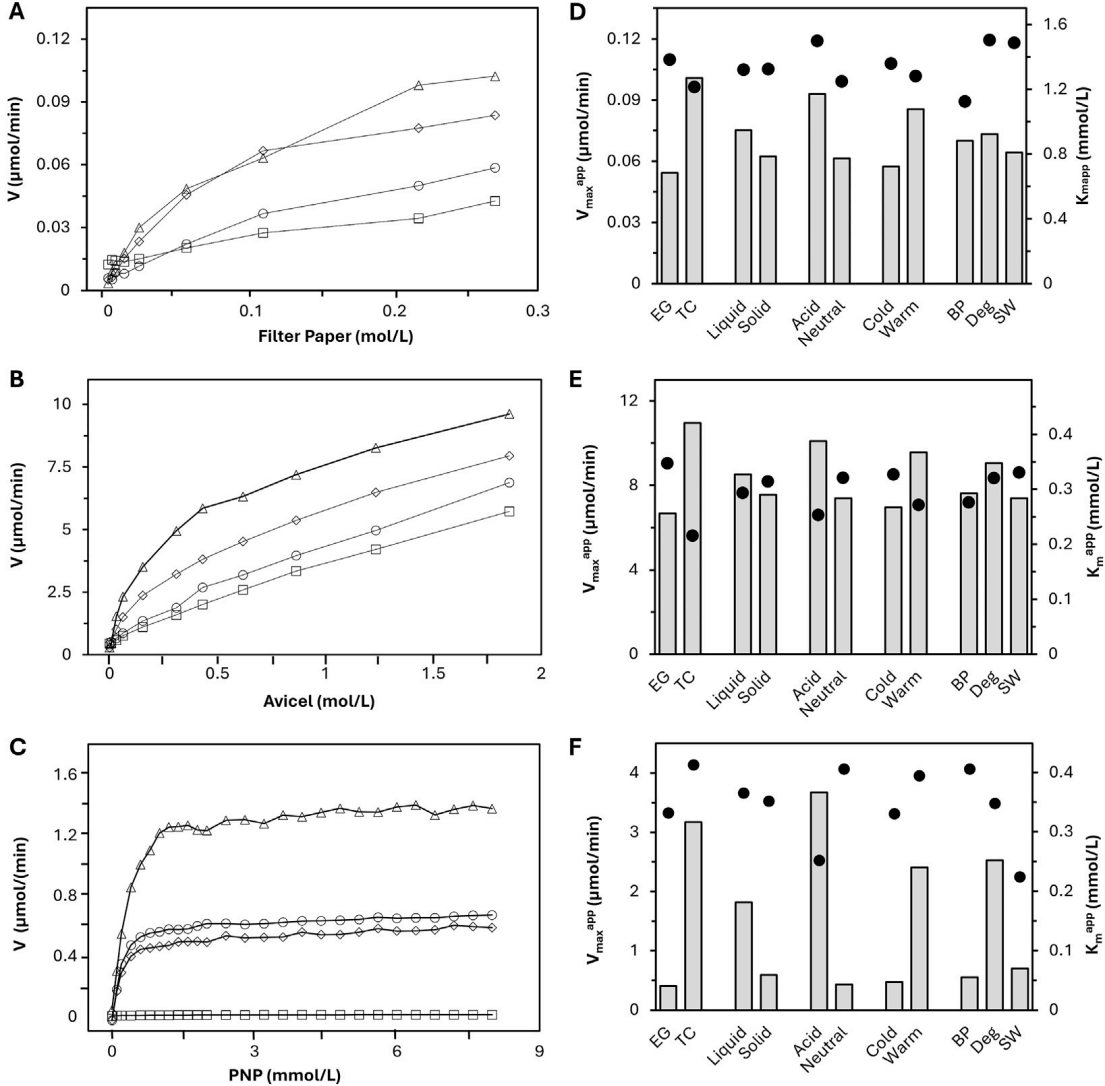
On the left are curves of reaction rate vs. substrate concentration from the filter paper **(A)**, Avicel **(B)**, and beta-glucosidase **(C)** assays for selected formulations: Cel 33 (TC Deg, diamonds), Cel 26 (TC BP, triangles), Cel 28 (BP, EG, squares), Cel 15 (SW, EG, circles). On the right is a summary of average *V*_*max*_^app^ (bars) and K_m_^app^ (dots) values of the active (n = 17) enzyme formulations across different enzyme categories for the filter paper **(D)**, Avicel **(E)**, and beta-glucosidase **(F)** assays.

Filter paper, Avicel, and PNP assays were carried out for each formulation using a range of substrate concentrations and a standardized enzyme concentration to generate curves plotting reaction rate versus substrate concentration (Figure 3) and determine apparent kinetic constants for each formulation. Measurement of released reducing sugars by the neocuproine method was used for quantification of the filter paper and Avicel assays. The apparent K_m_ and V_max_ of each formulation (K_m_^*app*^ and V_max_^*app*^) were calculated based on the curves generated from the substrate loading testing. Generated curves from a few model enzyme formulations are displayed in graphs for the three assays (Figure 3, left), and the averages of the K_m_^*app*^ and V_max_^*app*^ values across categories are summarized in the right column of Figure 3. The average is reported without standard deviation because of the heterogeneity of the data sets, which are based on highly variable formulations. In order to compare the data sets across categories despite this variation, categories were averaged to a single point for qualitative comparison within assays.

Because each assay had a different mechanism, direct relationships of single data points between assays also cannot be compared. For example, the PNP assay was the only assay driven by a liquid/liquid interaction, which cannot be reasonably compared to the liquid/solid interaction in the Avicel and filter paper assays. Between the filter paper and Avicel assays, there were differences in agitation where the Avicel assay had additional steel balls, making its V_max_^*app*^ values innately higher. The relationship between these assays also demonstrates the importance of agitation in cellulase hydrolysis methodologies, which has been reported previously (Vasconcelos and Cavaco-Paulo, 2006; Egan et al., 2023). Despite the inability to compare data points directly because of mechanistic differences, there is room to observe trends within assay datasets and discuss similarities or differences in trend across assays. Specific data for each of the 10 selected enzyme formulations used in the reactor experiments is shown in Table 2. This table further includes their values for endoglucanase activity based on the Megazyme KCellG5 assay kit, which is a single point characterization of the endoglucanase activity. The degradation of the substrate to glucose is influenced by the different enzymes present in the cellulase formulation. The filter paper (FP) assay displays the overall synergistic activity of all three enzyme classes (endoglucanases, CBHs and BGs) on the standard substrate filter paper Whatman No. 1. The assay using Avicel is designed to mostly display the activity of CBH and BG on the highly crystalline substrate Avicel Ph 101 because endoglucanases are thought to be inactive in crystalline regions. Finally, the PNP test monitors the isolated activity of BGs.

**TABLE 2:** Summary of categorization and assay data for the 10 formulations tested in the reactor (boxes in dark grey are below average for K_m_^app^ (higher substantivity) and above average for *V*_*max*_^app^ (increased reaction rate) while boxes in white are the opposite; boxes in light grey are approximately average). (Abbreviations: TC, total crude; EG, endo-enriched; L, liquid; S, solid; N, neutral; A, acid; W, warm; C, cold; DG, Degradation; BP, biopolishing; SW, stonewashing).

Enzyme	TC/EG	Solid/	Acid/	Cold/	Use case	FP		KCellG5	Avicel		BG	
						K_m_^*app*^ (mmol/L)	V_max_^*app*^ (µmol/min)	kUnits/mL	K_m_^*app*^ (mmol/L)	V_max_^*app*^ (µmol/min)	K_m_^*app*^ (mmol/L)	V_max_^*app*^ (µmol/min)
		Liquid	Neutral	Warm								
Cel 34	EG	L	N	W	DG	1.51	0.05	27.6	0.37	7.41	0.36	0.53
Cel 28	EG	L	N	W	BP	0.84	0.04	19.2	0.35	6.53	1.09	0.01
Cel 15	EG	S	A	C	SW	2.06	0.07	33.5	0.36	7.81	0.22	0.51
Cel 14	EG	S	A	C	SW	0.91	0.06	5.5	0.34	6.98	0.22	0.47
Cel 35	EG	L	N	W	DG	1.75	0.06	10.2	0.49	7.29	0.32	0.61
Cel 13	EG	S	N	C	BP	1.67	0.06	72.6	0.14	7.18	0.96	0.25
Cel 23	TC	S	N	C	BP	0.99	0.05	75.1	0.3	6.76	0.58	0.37
Cel 33	TC	L	A	W	DG	1.61	0.1	54.1	0.21	8.65	0.31	0.6
Cel 26	TC	L	A	C	BP	1.66	0.12	82	0.15	10.3	0.23	1.42
Cel 9	TC	L	A	W	DG	1.27	0.12	202	0.25	16.8	0.27	14.95

In the FP and Avicel assays, two categories of kinetic curves were observed: hyperbolic curves that increased up to a saturation point before slightly leveling off and straighter curves that continuously increase. The hyperbolic curves were attributed to TC formulations, where all three enzyme activities act synergistically together. In contrast, straight lines were attributed to endo-enriched (EG) formulations, which have only minor CBH activity. This synergistic activity means while the endoglucanases are cleaving chains, the concentration of chain ends on the substrate increases, granting the CBHs more active sites from which they release reducing sugars, ultimately resulting in a higher release rate. At some point, the number of active sites starts to outnumber the enzyme concentration, leading to the observed leveling off effect. Because the reaction rate continues to increase with more substrate even after the start of the plateau region, these curves were not typical of standard MM-kinetics, which is due to the constantly evolving substrate and multicomponent enzyme activities.

This linear relationship between substrate concentration and reducing sugar release rate could possibly be explained by endoglucanases randomly cleaving cellulose in the middle without separating the cleaved chain from the remaining cellulose fiber (Teeri, 1997) and a poor endoglucanase-to-cellobiohydrolase synergism in the formulation, leading to a lower production of reducing sugars (Luciano Silveira et al., 2012; Thoresen et al., 2021). Thus, for cellulase assay methods quantified by reducing sugar release alone, the CBH is the limiting factor, making TC formulations display in more hyperbolic curve geometry. Based on this principle, the authors have assigned formulations to TC and EG as a form of categorization.

The characterization of beta-glucosidase resulted in more reasonable kinetic data since it has the unique ability among cellulase assays to isolate one enzyme activity, specifically in liquid state. It is worth noting that some of the, EG formulations (e.g., Cel 15) showed higher beta-glucosidase activity than some formulations optimized for degradation and further categorized as TC (e.g., Cel 33) whereas other, EG formulations (Cel 28) showed no beta-glucosidase activity at all (Figure 3C).

The V_max_^*app*^ characterizes the potential hydrolysis speed, which followed similar trends across all assays within this dataset. TC showed higher activity than, EG; acidic formulations had a higher V_max_^*app*^ than neutral; warm were higher than cold; and liquid showed improved speed to solid. The differences were most pronounced for the PNP assay results. The findings between TC/EG and acid/neutral can be correlated since half of the TC formulations are acid while, EG formulations are about 80% neutral. Results from use case categorization showed similar V_max_^*app*^ trends in FP and Avicel assays where Deg had a slightly improved V_max_^*app*^ to BP and SW formulations. In the PNP assay, the average V_max_^*app*^ of Deg was almost 5 times higher than the BP and SW results.

The K_m_^*app*^ value is the substrate concentration at the half-maximum hydrolysis speed. A lower value indicates lower substrate concentration is needed to reach high reaction rates, implying higher substantivity between the enzyme and substrate. In the FP assay, K_m_^*app*^ shows minimal differences across categories compared to the other assays. Slight reductions were observed in the TC, neutral, and warm categories versus their respective counterparts. BP had a lower average K_m_^*app*^ value than Deg and SW, which were comparable to each other. In this dataset, seven out of the eight BP formulations were neutral, so it makes sense that the BP showed this reduced K_m_^*app*^ value.

The average K_m_^*app*^ values from the Avicel assay showed TC, acid, warm and BP with lower K_m_^*app*^ values in their categories. Compared to the FP assay, the K_m_^*app*^ more directly shows CBH substantivity to the crystalline substrate versus wholistic substantivity of endoglucanase and CBH in the filter paper assay. Since CBH proteins are thought to be more present in acidic formulations (60% of acidic formulations were TC in this work), this difference in trend between the two assays makes sense. For neutral, EG formulations, there is more opportunity to adhere to the filter paper than with the crystalline Avicel substrate, leading to lower K_m_^*app*^ values with the filter paper. However, since formulations that showed a straight line in the tests were attributed to EG, the results regarding K_m_^*app*^ need to be interpreted with care, since these tests did not reach their saturation point. In the PNP assay, the TC, warm, and BP groups had higher K_m_^*app*^ values within their category. Acid and SW formulations both exhibit the lowest K_m_^*app*^ values, which can be correlated in this dataset since both SW formulations are in the acidic category.

### 3.4 Hydrolysis performance in reactor

Ten formulations were selected to determine their performance in the reactor experiments for separation of cotton from polyester of blended textiles. Cel 9 (TC Deg) and 26 (TC BP) were chosen because they had the highest activities in every single activity assay carried out (Table 2). Cel 13 (EG BP) was chosen because it showed above average activity in the CellG5 and Avicel assays, so it was useful to see the importance of endoglucanase and CBH activity on total weight loss performance in the reactor. Cel 14 (EG SW) was selected because of its especially high demonstrated substantivity to the filter paper substrate (despite having below average activity with this assay) and low CellG5 assay activity, which is 2x lower than the next closest reactor-tested formulation. Cel 15 (EG SW) was chosen as a counter to Cel 14 as in the filter paper assay, Cel 15 showed notably low substantivity to the filter paper substrate yet showed improved activity to Cel 14 in the reactor experiment. Cel 23 (TC BP) was chosen because of its classification as a TC solid formulation, which differentiated it from other selected formulations. It also showed a high substantivity to the filter paper and high CellG5 activity. Cel 28 (EG BP) was one of two formulations which showed near-zero PNP activity, and it was chosen out of those because of its especially high FP substantivity, despite its below average performance in all other assays. Cel 33 (TC Deg) performed at or above average in every assay category, so its selection was based on its classification as a TC degradation formulation. Cel 34 (EG Deg) and 35 (EG Deg) were selected because they showed near average performance in every assay (except CellG5, which were well below average), so they were useful to use as a point for testing the all-around separation performance of an “average formulation” in this fiber separation use case.

**FIGURE 4:**
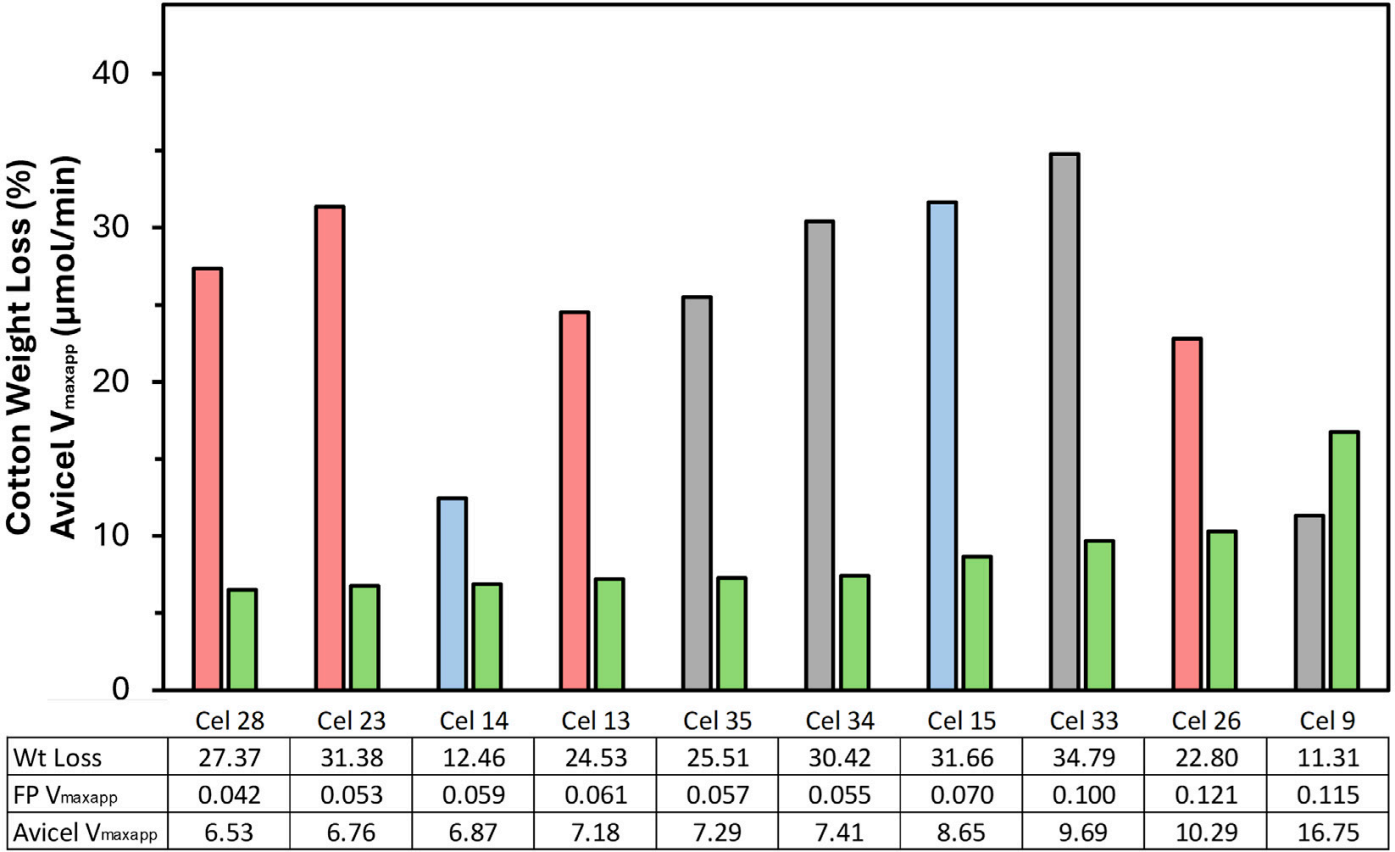
Weight loss of fabrics in the reactor after 4 h of enzyme treatment with different formulations at the same dosage. Sorted from left to right with increasing Avicel activity (*V*_*max*_^app^), shown in green. Weight Loss columns–Red: BP formulations; Grey: Deg formulations; Blue: SW formulations. Note that all hydrolysis data is a single point measurement except Cel 28.

Weight loss was determined gravimetrically based on the fabric weight before and after enzymatic treatment in the reactor, which was quantified in terms of cotton weight loss, based on the original cotton weight (50% of the total fabric weight). The weight loss data of the selected formulations is sorted from left to right in Figure 4 according to their Avicel V_max_^*app*^ value. Weight loss results from 11% to 35% were measured after 4 h in the reactor with the different formulations. One limitation within this part of the work is that all hydrolysis experiments were performed as single measurements, so no statistical analysis could be performed with the exception of Cel 28, which underwent repeated testing during method verification. Its performance is reported as its average from ten repetitions: 27.37% cotton removal with a standard deviation of 0.60%.

The weight loss observed did not correlate with the FP or Avicel V_max_^*app*^ results. Within the five worst assay performers, four were high performing (>25% weight loss) in the reactor, while the two formulations that performed best in the assays were among the three weakest in the reactor. Further, no clear trends could be established between most category distinctions, where there was no clear difference between TC/EG, cold/warm, or liquid/solid. Neutral enzyme formulations all had weight loss results between 25%-35% while the acidic formulations were mostly between 11%-25% with one outlier at 32%. A minor trend can be identified as it relates to the use case as BP and Deg on average both outperformed SW overall but showed no major difference from each other.

For the sake of uniformity in all tests, all reactor experiments were carried out at pH 6 °C and 60 °C, despite the supplier datasheet recommending a more acidic pH for some formulations. Therefore, one acidic formulation was tested at pH 5 °C and 60 °C and one cold formulation at pH 6 °C and 30 °C (data not shown). The acidic formulation Cel 9 (TC Deg) performed better at pH 5, increasing from 11.3% in neutral to 14.1% in acid (25% increase). The cold formulation Cel 14 (EG SW) performed better in warm conditions, showing a 21% increase in weight loss (from 10.3% to 12.5% in warm conditions).

**FIGURE 5:**
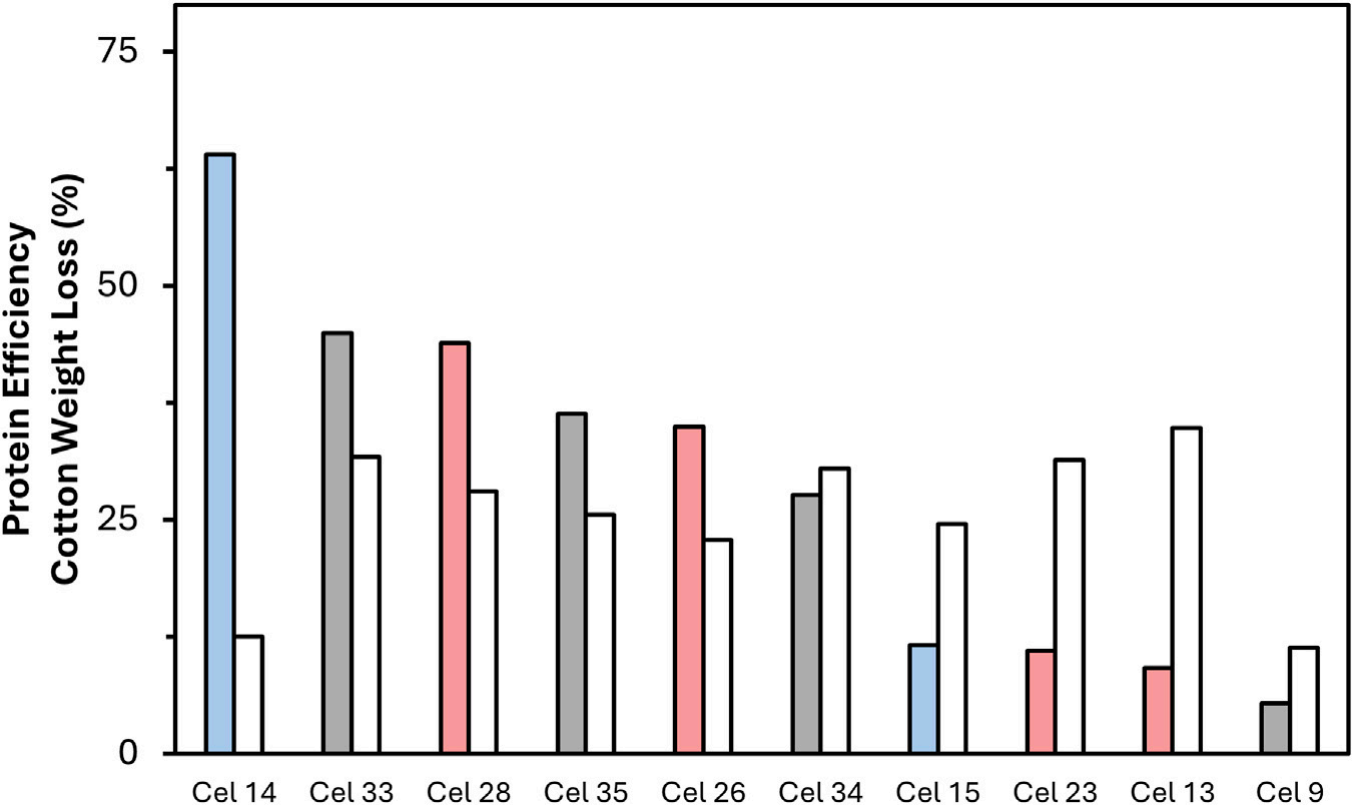
Protein efficiency and weight loss (%) for formulations tested in the reactor. Formulations presented in order of decreasing protein efficiency, which is color coded by use case (red = BP, black = Deg, blue = SW). The corresponding weight loss is shown in the white bar on the right. Note that all hydrolysis data is a single point measurement except Cel 28.

A limitation of the weight loss parameter was that it was based on the same dosage (20 g/kg fabric) across all formulation and therefore does not necessarily represent a wholistic picture of the formulations, so a further parameter is presented alongside weight loss in Figure 5. Because each enzyme formulation has a different protein concentration, the weight loss data was normalized to percent weight loss per gram of protein based on the expected concentration from the Bradford assay results. This value could be interpreted similarly to specific activity, which is based on the measured activity normalized by protein content. In this paper, it is discussed as “protein efficiency” or how effective the protein content was at achieving high weight loss in the system and is reported as a unitless value.

Interestingly, while Cel 9 (TC Deg) showed the lowest protein efficiency and the lowest weight loss value, Cel 14 (EG SW) showed the highest protein efficiency despite having the second lowest weight loss performance. Additionally, four high weight loss performing enzymes showed highly variable protein efficiency values. Cel 33 (TC Deg) and Cel 28 (EG BP) both showed high protein efficiency while Cel 13 (EG BP) and Cel 23 (TC BP) showed low protein efficiency. As with the weight loss results, the protein efficiency also showed no dependence on use case, TC/EG, or neutral/acid distinctions. Warm formulations showed slightly improved values to cold overall, where three of the five cold formulations had very low protein efficiency.

## 4 Discussion

### 4.1 Beyond shelf life vs. active enzymes

Enzyme formulations beyond their shelf life had the lowest amount of active protein, indicating their protein expectedly denatured over time. These formulations were tested out of curiosity for their extended life potential, so they were not expected to show high enzymatic activity. This was especially prevalent in solid formulations, which is relevant because solid enzymes have culturally been associated with increased power and shelf stability compared to liquid formulations. However, most solid foundational formulations showed extremely low active protein content, and all 5 of the excluded enzyme formulations that showed no activity were solid formulations. Further, within the active formulations, solid showed slower reaction rates than liquid in the assay screenings, so this cultural association may not be representative of the capability of commercial formulations. Unlike liquid formulations that are homogeneous, solid enzyme formulations can create significant local concentration differences of certain components. These high concentration zones may lead to physical contact between proteins and additives like crystalline buffer salts, potentially causing protein degradation that is not observed in liquid dispersions.

The V_max_^*app*^ values across the different categories were similar for active and beyond shelf life formulations in the filter paper and Avicel assay. In the PNP assay, the V_max_^*app*^ values from the beyond shelf life TC and acid formulations are reduced by a factor of 5 compared to the active formulations. The K_m_^*app*^ values for beyond shelf life formulations are generally comparable to the active formulations in the FP and Avicel assays and up to 4 times higher in the PNP assay (data not shown). Because of this relationship, it could be speculated that endoglucanases are more stable than CBHs, while BGs are the least stable cellulase component. However, since these formulations were not further characterized in terms of their activity loss over time, these results may not be representative of the whole picture and would require more study.

### 4.2 Formulation characterization

It is well known that the reaction rate of enzymes is influenced by the pH and temperature of the surrounding system in which they are active. In this work, acidic formulations are defined as those having optimal pH below 6 and neutral formulations are those between a pH of 6 and 7, as reported by the manufacturer. Of the determined, EG formulations, 9 were neutral and 2 were acidic whereas for the TC formulations, 3 were neutral and 3 were acidic. Given the skew of EG formulations to have optimal neutral conditions, it seems that in general, endoglucanases are optimal in more neutral conditions while CBHs—which are more present in TC—are then more optimal in acidic conditions. This relationship generally correlates TC with acidic and EG with neutral, which explains why acidic formulations outperform neutral in general in the assays. The assays are quantified by reducing sugar release, for which CBH proteins are the main driver while endoglucanases do not release reducing sugars until extended reaction times (Luciano Silveira et al., 2012). Therefore, TC formulations would show higher rates of reducing sugar release than EG formulations.

The cold classification was tested to see whether it is a technical requirement (i.e., it is the true optimal temperature of the enzyme) or is rather used as a label promoting economic benefit through reduction of heating costs. The “cold” label could be based on the principle that while some formulations have optimal activity in higher temperature, they still show substantial activity at lower reaction temperatures, which is beneficial for cold processing. Within this period of study, a cold formulation (Cel 14, EG SW) was tested at both warm (60 °C) and cold (30 °C) conditions, yielding higher levels of hydrolysis in the reactor system at elevated temperature despite its recommended use temperature being lower. This data suggests that those formulations marketed as cold by manufacturers have activity profiles that peak at elevated temperatures but still have notable activity at lower temperatures.

In contrast, optimal pH is known to be a technical parameter, so it was expected that the acidic formulations would perform better in acidic pH conditions, which was supported in this work. Processes under neutral conditions are preferred in several industrial applications to reduce chemical input for buffers.

### 4.3 Cellulase assays

Plotting reducing sugar release with increasing substrate concentration from the filter paper, Avicel, and PNP assays showed different kinetic curve geometries, including hyperbolic curves and more linear curves. It is generally recognized that cellulase formulations are too complicated for simple analysis according to standard MM kinetics because of formulation complexity with several different enzyme classes and substrate transformation throughout the reaction. This complexity generated curves like those seen in the Avicel assay, where saturation of the enzyme on the substrate may have never been reached, with curves that did not reach a V_max_^*app*^ value, although the substrate concentration was very high (up to 150 mg/mL). The PNP assay was more indicative of typical MM curves which reached a saturation point after which the activity no longer increased. It is attributed to its unique protein-targeting ability—wherein the BGs are not influenced by the cascade of the other components—and liquid/liquid reaction between enzyme and substrate.

It has been previously shown that TC formulations, being those that contain significant proportions of each cellulase component, have a higher rate of reducing sugar release using the filter paper assay than EG formulations (Luciano Silveira et al., 2012). The CBH component of a cellulase mixture is the one that is most responsible for reducing sugar generation, as its mechanism yields cellobiose. The reduced release rate from EG formulations is related to the mechanism, since reducing sugars are predominantly released by CBHs. Since endoglucanases randomly cleave chains, it could be that some short fragments can be detected as reducing sugar, far less than for CBHs which always release reducing sugars. In the present results, linear curves are associated with endo-enriched formulations, having a low level of CBH, while those with hyperbolic shape correspond with TC formulations. This distinction was used as an additional category for enzyme classification throughout the work and was helpful for understanding the formulation behavior and relationships between the categories. Because TC formulations outperformed EG formulations on average in all assays, including the endoglucanase-targeting CellG5 assay, it indicates that the content of endoglucanases is not increased in EG formulations, but rather that the CBH content is reduced.

Due to their use case and required mode of action, it might be expected that BP and Deg formulations would be TC rather than endo-enriched, requiring an acidic pH and elevated temperature. Although technical datasheets indicate that formulations are for BP and Deg use, some showed linear curves in the assays and were therefore assigned to the EG category. Specifically, only three of the eight BP formulations and only three of seven Deg formulations were TC. On the other hand, most of the EG classified formulations had a neutral optimal pH according to the datasheets, which supports the findings in view of the general optimal pH requirements for endoglucanases.

Aside from this, the results from the assay screening for the different use cases were able to be correlated with the known mechanisms of each case. For example, during a stonewashing process, the mechanistic goal is to induce surface peeling of the cellulose fibers (or yarns) to remove indigo dye particles from the surface, leaving a color effect on the fabric since the yarn is surface dyed. Knowing this, it made sense that the SW formulations had lower activities in general to avoid excessive fiber damage and performed according to the determined EG kinetics in the filter paper assay. Biopolishing is intended to cleave fibers from the fabric surface to leave a smoother substrate, and BP formulations performed highly in all assays except the PNP assay, which is what distinguishes them from Deg formulations. The main intention of Deg formulations is to ensure high yield of a highly pure glucose product for fermentation into new materials, so they have high activities in all assays and extremely improved BG activity to the other two use cases. This mechanism more closely mimics what cellulase proteins in nature do—generate a constant glucose output as a source—which is not needed for the new use case of separating cotton from blended textile waste.

### 4.4 Reactor

Our findings highlight a weak correlation between enzymatic assays and reactor performance in weight loss production. Cel 9 (TC Deg) and 26 (TC BP) displayed high substantivity to the model substrates and consistently high reaction rates in the cellulase assays (Table 2). Despite these indicators, Cel 9 showed the lowest reactor results of the selected formulations and Cel 26 was below average (Figure 4). Because the formulations were dosed based on volume rather than protein content, they were further normalized to weight loss per Gram of protein. In this parameter, Cel 9 remained the lowest value, while Cel 26 had less protein in it, so showed higher protein efficiency than Cel 9 with this calculated value (Figure 5). The reduced reactor performance of these two formulations could be attributed to the adsorption of some proteins onto PET instead of cotton as a competing mechanism, but that was not further investigated within this work.

In the reactor, neutral formulations outperform the acidic ones regarding weight loss. Notably, Cel 9 and 26 are acidic formulations, and the use of Cel 9 in its optimal pH did yield improved weight loss results (a 25% increase), though it still did not meet the other tested formulation results (data not shown). In general, the discrepancy between neutral and acid results could be an artifact of the neutral testing conditions, in which acidic formulations are known to be working at a non-optimal pH.

The best performer for weight loss was Cel 13 (EG BP), which showed below average performance in the filter paper and PNP assays but above average performance in the CellG5 and Avicel assays. Cel 34 (EG Deg) was also a high performing formulation, which showed below average performance in all assays except PNP, where it was average. Given these relationships, it is apparent that the assay screening and its quantification by reducing sugar release does not correlate with observed weight loss production, which is ultimately quantified by a different mechanism.

When deciding which commercial formulations are most important for future research in this area, it was deemed important to emphasize a balance between weight loss production and protein efficiency, since protein efficiency also relates to the overall costs of the process. Since the prices of the formulations were not evaluated in this work, we refer to efficiency. Because of that, Cel 14 (EG SW, which had the highest protein efficiency value) was not favorable because of its low weight loss production and Cel 13 (the highest for weight loss production) was not favorable because of its low protein efficiency. Cel 33 (TC Deg) appears to be the best performer in terms of balance under these conditions with weight loss production above 30% and the second highest protein efficiency. The next 3 most balanced formulations were Cel 28 (EG BP), Cel 34 (EG Deg), and Cel 35 (EG Deg). The propensity of EG formulations to yield balanced formulations in the reactor experiments could indicate that endoglucanase proteins are the most influential for an efficient separation scheme. Interestingly, all four of these formulations were at or below average performance in the CellG5 assay, suggesting that assay is also not a good indicator for reactor performance.

**FIGURE 6:**
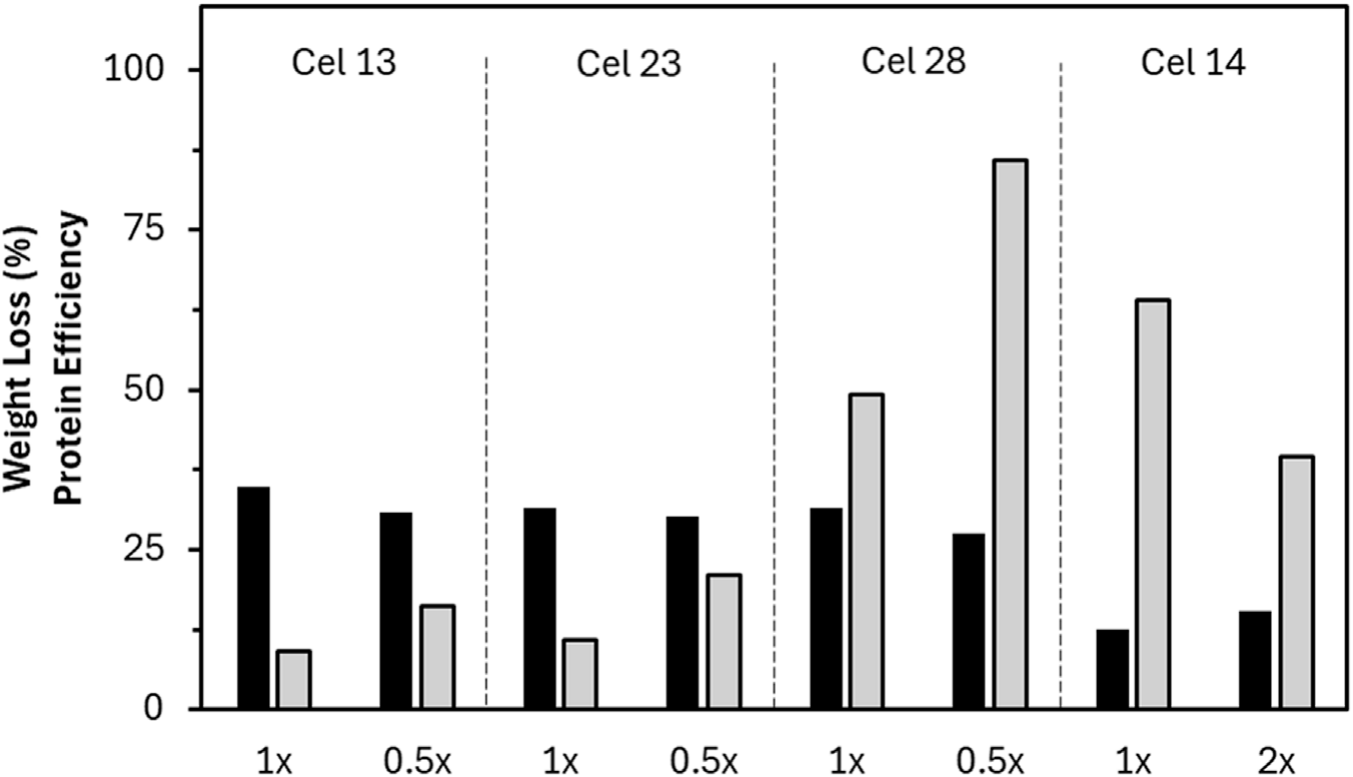
Comparison of selected enzymes tested with either halved (0.5x) or doubled (2x) enzyme content and the resultant weight loss (black) and protein efficiency (grey) values compared to the standard dose (1x). Note that all hydrolysis data is a single point measurement except Cel 28.

Interestingly, Cel 13 (EG BP) and 14 (EG SW) come from the same manufacturer but have completely different performances. Cel 14 showed high protein efficiency with low weight loss, while Cel 13 showed the highest weight loss overall but low protein efficiency, since Cel 13 had the highest protein concentration of all reactor formulations. Because reactor dosage was set to a standard based on volume, it seemed likely that the dosage was already beyond the kinetic plateau for Cel 13, artificially reducing the protein efficiency value because of overdosing. Hence, the specific concentration of Cel 13 was investigated to check for overdosing by conducting the experiment at half of the standard dose. The weight loss results were only slightly reduced (34%–31%), leading to a higher protein efficiency value (Figure 6). This idea was also investigated with Cel 23 (TC BP) because it similarly had high weight loss and low protein efficiency at the standard dosage. The same effect was observed where at the half dose, the weight loss showed minimal reduction leading to an increase in protein efficiency. Because Cel 28 (EG BP) has high weight loss and high protein efficiency, it was additionally tested in the reactor at reduced concentration to investigate its protein efficiency value. Similar to Cel 13 and 23, the half dose resulted in similar weight loss and therefore improved protein efficiency.

In contrast, Cel 14 had the lowest protein concentration of the reactor formulations, which could have artificially increased the protein efficiency value. It was further tested with a doubled enzyme dosage, showing a weight loss improvement (from 12% to 15%), leading to a decrease in calculated protein efficiency. Doubling the dosage did not lead to a sharp increase in weight loss, meaning that the standard dosage was already near the end of the steep increase region. Although the high protein efficiency is maintained, further increases in dosage would not lead to substantial weight loss improvement.

Since acid formulations were tested under neutral conditions and their performance possibly would be 25% increased, the CBH contribution may not be fully represented in the data. Despite this, an acidic formulation yielded the best performance. Complementary to endoglucanases as the main contributors to weight loss, CBHs are synergistically important. Weight loss is generated faster by breaking away big particles (facilitated mainly by endoglucanases) with support from CBHs that peel away the newly created ends, making space for the endoglucanases to penetrate the fiber more. From the high performance of the acidic Cel 33, it was demonstrated that CBH played an important role in weight loss. The three best performing reactor formulations had considerable Avicel activity, but none of them were above average. Because it is known that cellobiose can lead to enzyme inhibition, the presence of beta-glucosidase would be favorable to support the CBH activity.

### 4.5 SDS-PAGE

**FIGURE 7:**
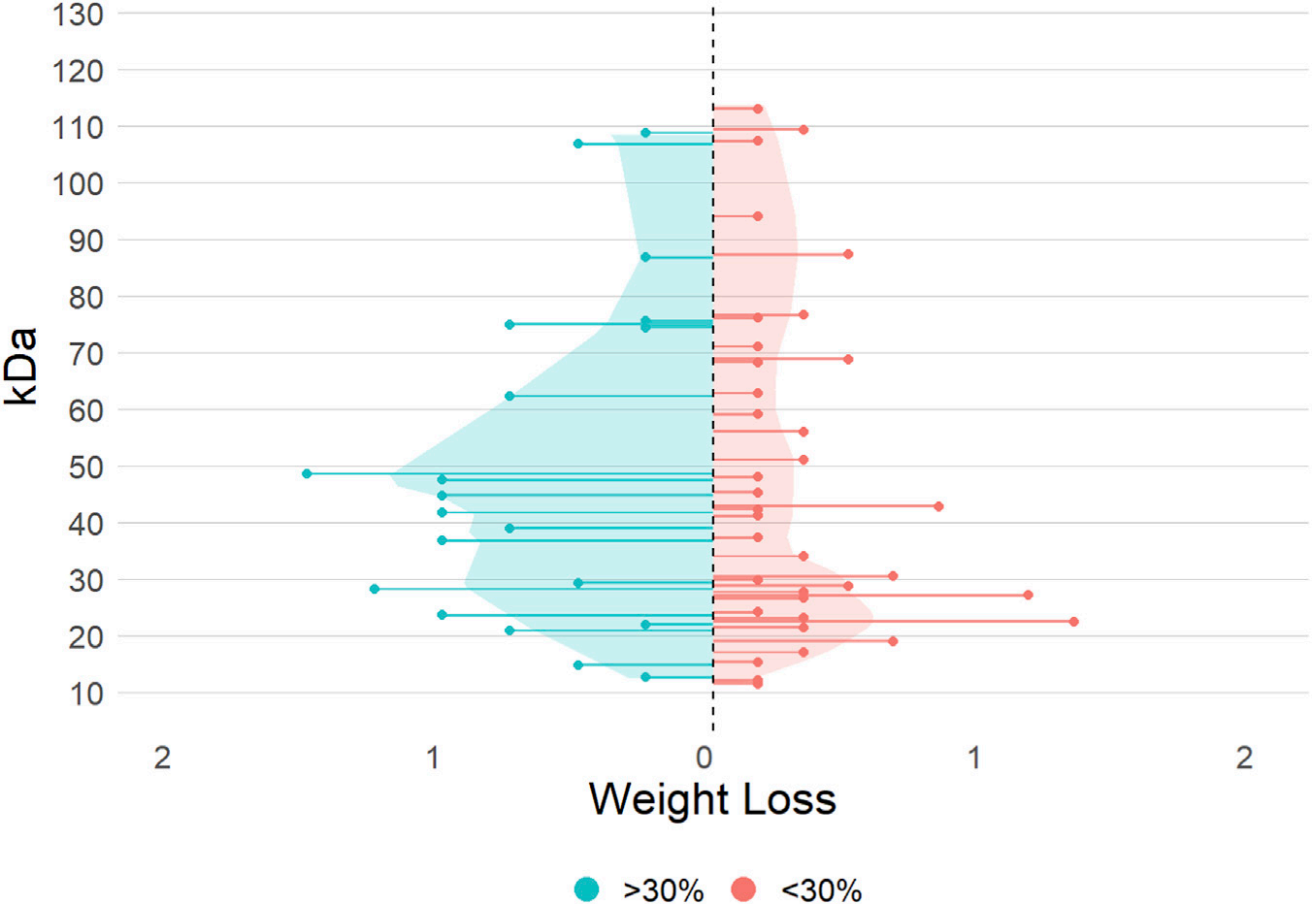
Average band intensity evaluated from the SDS-PAGE data of the 10 reactor formulations, categorized by low (orange, <30%) and high (blue, >30%) weight loss performance. Shaded areas under LOESS-smoothed curves (span = 0.4) highlight the overall signal distribution per group across the molecular weight range.

The SDS-PAGE data was further used to look at commonalities between different groups of formulations based on average band intensity for a specified subset of enzymes and speculate on the band associations with proteins using previous analysis from other groups (Supplementary Figure S2). Special interest was placed on formulations which had especially high weight loss production (defined as >30%). Some of the prominent peaks that are unique to this group of formulations are 29, 37, 45–49, and 62 kDa. These peaks could correspond with EGV (Tolan and Foody, 1999), EG (Nisar et al., 2022), EGII (Kyriacou et al., 1987; Hu et al., 2010), and CBHII (Goyal et al., 1991; Hu et al., 2010). While we cannot clearly attribute TC or endo-enriched to perform better in the reactor regarding weight loss, these bands appear to be important for achieving high weight loss in both species. Peaks unique to the formulations with <30% weight loss production (Figure 7) include 22 and 27 kDa—which have previously been identified as EGIII (Tolan and Foody, 1999) and, EG (unspecified) (Chen et al., 1997)—and 69 kDa, which has been associated with CBHII previously (Tolan and Foody, 1999). This indicates that not all EG proteins are similarly important in this kind of separation methodology, which could inform future engineering of formulations in this kind of mechanism.

Because of the high protein efficiency of the Cel 14 (EG SW) formulation, the bands in its SDS-PAGE analysis were compared to the other reactor formulations as possibly being highly significant for fast separation. It had six bands with different intensities: 11 (low), 22 (high), 27 (high), 43 (low), 67 kDa (low), and 94 (low). 22 and 27 kDa were the only bands in Cel 14 with high intensity, so it could be assumed they are the main contributors to its protein efficiency. At 22 kDa, five out of the ten reactor formulations had a band, though it was the most intense in Cel 14. Cel 28 (EG BP) was the only other formulation to have a band at 27 kDa, with a notably very high intensity. Cel 28 also had high protein efficiency in the reactor with only three bands: 12, 22, and 27 kDa without showing any beta-glucosidase activity, suggesting that it is an endoglucanase-only formulation and emphasizing the role of bulk hydrolysis by endoglucanases for good separation efficiency. This finding is supported by the relatively low and straight Michaelis-Menten curves in the activity assays for Cel 28 versus the high weight loss in the reactor experiments.

Importantly, SDS-PAGE can only provide assumptions about the enzymes corresponding to each band, so the results should be interpreted with caution. Precise protein identification can be achieved through additional analysis like combining SDS-PAGE (one or two dimensional) with MALDI-TOF (Matrix-Assisted Laser Desorption/Ionization Time-of-Flight) which was out of scope in this work.

## 5 Conclusion

A comprehensive characterization of 35 commercial enzyme formulations with a focus on 17 active formulations was conducted through various assays: protein and reducing sugar content and cellulase component-targeting assays. The performance of ten formulations was further tested in reactor-based hydrolysis experiments. Notably, the reactor experiments presented were single point measurements, so there are some limitations to the conclusions. Trends between their activities and known classifications could be observed, and conclusions about the nature of cellulase enzymes and their behavior were drawn.

Enzyme kinetics data was derived from the filter paper, Avicel, and PNP assays and compared based on known formulation classification like physical state, use case, and optimal temperature and pH. The formulations were further categorized as being total crude (TC) or endo-enriched (EG) based on their assay response. Significant variation was observed in protein concentration across formulations, with TC formulations and solid enzymes generally exhibiting higher protein content than their EG and liquid counterparts. In general, similar trends across all assays within this dataset were observed. TC showed higher activity than EG; acidic formulations had a higher V_max_^*app*^ than neutral; values for warm formulations were higher than for cold; and liquid showed improved reaction rate to solid. Optimal pH of the formulation and the cellulase components were associated: endoglucanases with neutral and cellobiohydrolases (CBH) with acidic optimal pH. Minimal differences between the use cases (biopolishing, stonewashing, and degradation) were observed, except in the PNP assay where formulations for degradation showed much higher beta-glucosidase activity on average because they are intended to generate high yields of glucose. The results from the activity assays did not correlate with the reactor experiments, but they offered some helpful insights to understand the differences between the cellulase component reaction mechanisms.

Though many were unbalanced, some formulations showed both high weight loss and protein efficiency in the reactor. No specific correlation between EG and TC with either weight loss or protein efficiency was found. However, the formulations that showed the most balanced response (high weight loss and high protein efficiency at the current dosage) tended to be EG. This relationship suggests that the bulk hydrolysis by EG proteins on the substrate is one of the driving factors behind effective weight loss production for a heterogeneous substrate. However, the synergistic effect of CBH proteins should not be underestimated since the best performing formulation was TC. The need for CBH also consequently requires the addition of some BG proteins to avoid cellulase activity inhibition by cellobiose. These findings, while primarily investigated for the purpose of textile blend separation can also be extended to other areas in which cellulase activity is useful, perhaps being able to inform which activities are important for different applications.

Overall, this study provides insights into the enzymatic hydrolysis of blended textiles, emphasizing the need for tailored enzyme formulations designed specifically for fiber separation applications. A general recommendation to use formulations for biopolishing, stonewashing or degradation is not possible. Furthermore, the findings underscore the importance of selecting enzymes not solely based on conventional activity assays but also considering reactor performance and protein efficiency metrics. In summary, bulk separation in agitated reactors is highly influenced by endoglucanases for successful separation of cotton and polyester textiles since the focus of the separation mechanism is to release large cellulose fragments rather than degrade crystalline parts entirely. Therefore, for this novel use case, CBH activities are complementary to endoglucanase activity, but they do not necessarily need to be highly concentrated. Finally, though beta-glucosidase proteins do not seem to have a major influence on weight loss, a small amount of BG activity supports the overall hydrolysis. Given this, it is suggested that a formulation optimized for separation would include a high proportion of endoglucanase, moderate CBH activity, and a small portion of BG which is ultimately different from the biopolishing, degradation and stonewashing formulations available today.

## Data Availability

The raw data supporting the conclusions of this article will be made available by the authors, without undue reservation.

## Supplementary material

SUPPLEMENTARY FIGURE S1 Illustration of how SDS-PAGE bands were associated with their intensity.

SUPPLEMENTARY FIGURE S2 Representation of bands based on distinctions shown in Supplementary Figure S1 for each current formulation.
