# Supplementary figures and images for: Diving into commercial cellulase formulations for circular polyester/cotton separation through targeted depolymerization of cotton
Source: Front Bioeng Biotechnol. 2025 Sep 19;13:1632772. doi: 10.3389/fbioe.2025.1632772 (PMC12491305; doi:10.3389/fbioe.2025.1632772)

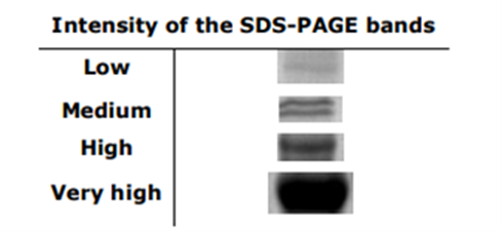

Supplement: Supplementary file 1 [file Image1.tiff]

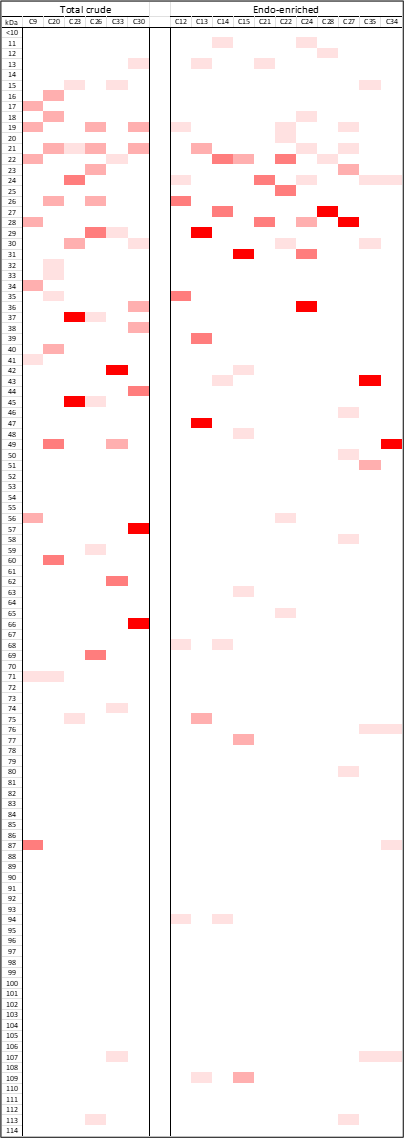

Supplement: Supplementary file 2 [file Image2.tiff]
